# COVID-19: challenges for a new epoch

**DOI:** 10.1590/0037-8682-0270-2020

**Published:** 2020-06-01

**Authors:** Cleyton Martins da Silva, Graciela Arbilla

**Affiliations:** 1 Universidade Federal do Rio de Janeiro, Instituto de Química, Rio de Janeiro, RJ, Brasil.; 2 Universidade Veiga de Almeida, Rio de Janeiro, RJ, Brasil.

Dear Editor:

The term “pan-epidemic Anthropocene” was proposed by Lucey et al. to refer to multifocal large infectious disease epidemics related to anthropogenic forces that have impacted and transformed the Earth System^1^. Smallpox was restricted to the Old World for thousands of years but became the first “pan-epidemic Anthropocene” when Europeans brought it to the Americas in the 16th century. 

The arrival of Europeans in America was considered as the first globalization event where the two civilizations met on unequal terms after 12,000 years of separation. Smallpox emerged in January 1519 and spread rapidly to the mainland of Central America. Native Americans had no immunity to the infections brought in by the Europeans. Smallpox, influenza, measles, typhus, pneumonia, scarlet fever, malaria, and yellow fever emerged subsequently and affected millions. Approximately 90% of the estimated 50-80 million Native American population died of these diseases, which suggests approximately 10% of mankind died during 1493-1650. This event initiated the globalization and homogenization of the world’s species and diseases^2^.

In the 20th century, epidemics and pandemics continued to kill millions of people. The first pandemic of the 21st century was reported as influenza A (H1N1) in 2009-2010 that resulted in 100,000-400,000 deaths in the first year; however, for the first time, a vaccine was developed, produced, and distributed worldwide during the first year^3^. 

Globalization, the exchange of species, materials, energy, and culture, as well as urbanization and the increase in the world’s population and the global flow of people, had a clear, and probably irreversible, impact on the environment and the equilibrium of the Earth System that led to the proposal of a new geological Epoch, called Anthropocene^4^. The term “Anthropocene” was proposed by Crutzen and Stoermer in 2000, to emphasize the central role of mankind in geology and ecology^5^. The increasing impact of human actions on the environment was substantial, global, and long-lasting^2^. There is an increasing consensus on the formal recognition of Anthropocene as a geological Epoch, functionally and stratigraphically distinct from the Holocene^6^. New anthropogenic materials such as plastics, concrete, aluminum, and synthetic fibers; modification of sedimentary process; wastes from nuclear weapons testing; increase in atmospheric methane and CO_2_ concentrations; changes in carbon, nitrogen, and phosphorus cycles; climate and biotic changes are clear Earth System trends that confirm the transformations to be driven by human residents. Globalization, urbanization, transportation, increase in economic activities, and the flux of people, live species, and manufactured products are clear socioeconomic trends that characterize this new epoch, commonly referred to as “Human Epoch.” 

All these factors, in addition to political destabilization and civil and international wars, led to poverty, increase in maternal and child death, and spread of diseases. Hotez^7^ reported that these changes have promoted the emergence of catastrophic neglected tropical diseases (NTDs), first being dengue fever in the 1980s in the American continent, chikungunya and Zika virus infections in Latin-American and Caribbean regions, and malaria in the Amazonian region of South America. NTDs along with other infections such as leishmaniasis, schistosomiasis, and Middle East Respiratory Syndrome (MERS) coronavirus infection, measles, and polio spread in the Middle East and North Africa. During 2014-2015, the Western African Ebola virus epidemic decimated numerous families and caused a socioeconomic disruption in Guinea, Liberia, and Sierra Leone.

The outbreak of COVID-19 provides strong evidence that urbanization and globalization have changed the way people live in communities, and advances in transport and communications have led to a rapid spread of diseases, through both domestic and international transportation modes such as buses, trains, boats, and flights. Increased density of people in residences, public transportation, work environments, shopping centers, and cultural, political, sport, and religious events have increased the possibilities of virus transmission in the metros. Social inequalities lead to higher risks, particularly in middle- and low-income countries that generally have weaker health systems and a limited capacity to handle a rapid surge in cases. Poverty contributes to disseminating epidemics and pandemics, while simultaneously helping to perpetuate poverty through their longstanding negative effects.

As no specific drugs or vaccines are available, the mitigation of the exponential spread of COVID-19 relies on community mitigation strategies^8^. There is a crescent consensus that social isolation to prevent the virus spread is the correct strategy from not only the human rights point of view but also the economic point of view. It seems clear that the collapse of the health care systems and millions of deaths would decimate countries financially, and as a society, thus saving human lives must be the governments’ first priority. 

Although the pandemic is a global phenomenon and the consequence of urbanization, transport, and interchange of people, live species, and manufactured products, its impact is greatly shaped by decisions taken by the individual governments. Many governments responded swiftly while others, after an initial slow response, acknowledged their error and adopted the World Health Organization’s (WHO) recommendations. Unfortunately for all, some are still ignoring WHO’s recommendations on avoiding mass gatherings. Recently, Croda et al.^9^ reported on the progression of COVID-19 cases in Brazil and previous experiences with other health emergencies that constituted an important legacy in dealing with epidemics and demonstrated Brazil’s scientific capacity. They also discussed the initially implemented measures to reduce the virus spread and mortality. Despite the exceptional efforts by the state governors and city mayors, the response of the country’s President contributes to the growing uncertainty among the population about the health risks and economic impacts of the pandemic^10^. In uncertain times, positive leadership navigates through a crisis; leaders are required to make the right decisions based on science and to craft a good narrative to clarify the problems and unite the population to manage the situation. Global problems require global solutions and everybody’s efforts. 

In addition to all the negative impacts, the pandemic has opened new opportunities, displayed examples of solidarity in the local communities, and allowed sharing of resources, information, and expertise from countries further ahead in the pandemic or with better results and knowledge in controlling the spread. The scientific communities worldwide have joined hands and many universities have organized groups of researchers and students to help in the pandemic relief efforts in every possible manner. 

The pandemic is a strong reminder that to be prepared for the future, a fundamental change in our mindset, commitments, and values is necessary. It is necessary to create a change in our current way of living and a concentrated effort to establish conditions for humanity to manage itself in the Anthropocene, the “Age of Humans.” British economist Kate Raworth, from Oxford University, has rightly mentioned that humanity’s 21st-century challenge is to meet the needs of all within the means of the Planet^11^. Her model, known as “Doughnut Economics”, has recently been adopted to guide Amsterdam out of the economic impact left by the coronavirus pandemic^12^. Environmental repair, renewable energy, more investments in science, public health, and education are needed to reduce inequalities, climate changes, and the human impact on Earth’s equilibrium, else, humanity will never be prepared enough to confront the devastating challenges of a pandemic. Humans have the opportunity of saving their own lives and the life of all species in the Earth System through advanced technology and science.

Preparedness is crucial to reduce the health, economic, and social impacts of a future epidemic, it is also the only way to avoid the spread of other diseases. Pandemics are not aleatory events but are the consequence of human interactions with the environment and could be avoided or reduced through science and investments in health, education and transportation and improved through better conditions of living. 

This is an opportunity for the global community to take advantage of the spirit of cooperation, embrace diversity and arrive at a necessary common global agreement to manage the future of Earth collectively.
